# Supplementary material for: N-acetylation of α-synuclein enhances synaptic vesicle clustering mediated by α-synuclein and lysophosphatidylcholine
Source: bioRxiv. 2024 Aug 21:2024.03.04.583437. Originally published 2024 Mar 6. Preprint. [Version 2] doi: 10.1101/2024.03.04.583437 (PMC10942363; doi:10.1101/2024.03.04.583437)
Supplement: Supplement 1 [file media-1.pdf]

Table S1 Identified cross-linked peptides of sample 1 between ^{15}N-Ac-α-syn and ^{14}N-Ac-α-syn in LPC (protein: lipid= 1: 50, mol: mol).

Score	Δ (ppm)	Res of Pr 1*	Sequence of Pr 1*	Res of Pr2*	Sequence of Pr 2*	Mass (Da)
1.43E-20	-0.67	12	$\mathrm{A^{11}\underline{K}EGVVAAAEK^{21}}$	23	$\mathrm{T^{22}\underline{K}QGVAEAAGK^{32}}$	2268.2324
5.94E-14	0.65	32	$\mathrm{Q^{24}GVAEAAG\underline{K}TK^{34}}$	10	$\mathrm{G^{9}LS\underline{K}AK^{12}}$	1799.0151
8.04E-13	-0.04	34	$\mathrm{T^{33}\underline{K}EGVLYVGSK^{43}}$	23	$\mathrm{T^{22}\underline{K}QGVAEAAGK^{32}}$	2376.2899
2.17E-12	-1.77	32	$\mathrm{T^{22}KQGVAEAAG\underline{K}TK^{34}}$	23	$\mathrm{T^{22}\underline{K}QGVAEAAGK^{32}}$	2484.3546
5.89E-12	0.46	45	$\mathrm{T^{44}\underline{K}EGVVHGVATVAEK^{58}}$	12	$\mathrm{T^{22}\underline{K}QGVAEAAGK^{32}}$	2733.4911
1.33E-11	0.22	12	$\mathrm{A^{11}\underline{K}EGVVAAAEK^{21}}$	97	$\mathrm{\underline{K}^{97}DQLGK^{102}}$	1897.0519
2.22E-11	-2.07	58	$\mathrm{T^{44}KEGVVHGVATVAE\underline{K}TK^{60}}$	10	$\mathrm{G^{9}LS\underline{K}AK^{12}}$	2493.4165
1.49E-10	-2.77	23	$\mathrm{T^{22}\underline{K}QGVAEAAGK^{32}}$	97	$\mathrm{\underline{K}^{97}DQLGK^{102}}$	1884.0315
2.94E-10	-1.25	34	$\mathrm{T^{33}\underline{K}EGVLYVGSK^{43}}$	12	$\mathrm{A^{11}\underline{K}EGVVAAAEK^{21}}$	2389.3103
1.42E-08	-1.10	21	$\mathrm{E^{13}GVVAAAE\underline{K}TK^{23}}$	10	$\mathrm{G^{9}LS\underline{K}AK^{12}}$	1842.0461
2.07E-08	-0.67	58	$\mathrm{E^{46}GVVHGVATVAE\underline{K}TKEQVTNVGGAVVTGVTAVAQK^{80}}$	12	$\mathrm{G^{7}LSKA\underline{K}EGVVAAAEK^{21}}$	5027.7505
3.43E-08	-2.37	43	$\mathrm{T^{33}KEGVLYVGS\underline{K}TK^{45}}$	10	$\mathrm{G^{9}LS\underline{K}AK^{12}}$	2149.2357
5.39E-07	2.62	43	$\mathrm{T^{33}KEGVLYVGS\underline{K}TK^{45}}$	43	$\mathrm{T^{33}KEGVLYVGS\underline{K}TK^{45}}$	2955.6531

Δ: mass error for crosslink assignments; Res: crosslinked residue (sequence number) within corresponding proteins; Pr: protein; Underlined K in sequence is the cross-linked lysine.

*The crosslinked pair of residues were from two individual Ac-α-syn: ^{14}N-Ac-α-syn and ^{14}N-Ac-α-syn, ^{14}N-Ac-α-syn and ^{15}N-Ac-α-syn, ^{15}N-Ac-α-syn and ^{14}N-Ac-α-syn, or ^{15}N-Ac-α-syn and ^{15}N-Ac-α-syn. Detailed data processing procedure could be referred to Methods session.

Table S2 Identified cross-linked peptides of sample 2 between ^{15}N-Ac-α-syn and ^{14}N-Ac-α-syn in LPC (protein: lipid= 1: 50, mol: mol).

Score	Δ (ppm)	Res of Pr 1*	Sequence of Pr 1*	Res of Pr2*	Sequence of Pr 2*	Mass (Da)
2.56E-17	-0.42	45	T$^{44}\underline{K}$EGVVHGVATVAEK^{58}	12	A$^{11}\underline{K}$EGVVAAAEK^{21}	2733.4911
3.92E-15	0.45	12	A$^{11}\underline{K}$EGVVAAAEK^{21}	23	T$^{22}\underline{K}$QGVAEAAGK^{32}	2268.2324
3.41E-14	0.97	34	T$^{33}\underline{K}$EGVLYVGSK^{43}	23	T$^{22}\underline{K}$QGVAEAAGK^{32}	2376.2899
2.82E-13	0.45	32	Q^{24}GVAEAAG$\underline{K}$TK^{34}	10	G^{7}LS$\underline{K}$AK^{10}	1799.0151
6.88E-13	0.59	43	T^{33}KEGVLYVGS$\underline{K}$TK^{45}	12	A$^{11}\underline{K}$EGVVAAAEK^{21}	2618.4529
4.51E-12	1.04	12	A$^{11}\underline{K}$EGVVAAAEK^{21}	97	$\underline{K}^{97}$DQLGK^{102}	1897.0519
2.59E-11	-2.90	23	T$^{22}\underline{K}$QGVAEAAGK^{32}	97	$\underline{K}^{97}$DQLGK^{102}	1884.0315
1.28E-10	0.36	32	Q^{24}GVAEAAG$\underline{K}$TK^{34}	97	$\underline{K}^{97}$DQLGK^{102}	1884.0315
1.07E-09	-1.42	21	A^{11}KEGVVAAAE$\underline{K}$TK^{23}	12	A$^{11}\underline{K}$EGVVAAAEK^{21}	1965.9903
2.81E-09	2.82	21	E^{13}GVVAAAE$\underline{K}$TK^{23}	10	G^{7}LS$\underline{K}$AK^{10}	2510.3954
7.27E-08	0.30	43	T^{33}KEGVLYVGS$\underline{K}$TK^{45}	97	$\underline{K}^{97}$DQLGK^{102}	1842.0461

Δ: mass error for crosslink assignments; Res: crosslinked residue (sequence number) within corresponding proteins; Pr: protein; Underlined K in sequence is the cross-linked lysine.

*The crosslinked pair of residues were from two individual Ac-α-syn: ^{14}N-Ac-α-syn and ^{14}N-Ac-α-syn, ^{14}N-Ac-α-syn and ^{15}N-Ac-α-syn, ^{15}N-Ac-α-syn and ^{14}N-Ac-α-syn, or ^{15}N-Ac-α-syn and ^{15}N-Ac-α-syn. Detailed data processing procedure could be referred to Methods session.

Table S3 Identified cross-linked peptides of sample 3 between ^{15}N-Ac-α-syn and ^{14}N-Ac-α-syn in LPC (protein: lipid= 1: 50, mol: mol).

Score	Δ (ppm)	Res of Pr 1*	Sequence of Pr 1*	Res of Pr2*	Sequence of Pr 2*	Mass (Da)
3.49E-15	0.21	12	A^{11}KEGVVAAAEK^{21}	23	T^{22}KQGVAEAAGK^{32}	2268.2324
1.32E-13	3.78	60	T^{59}KEQVTNVGGAVVTGVTAVAQK^{80}	34	T^{33}KEGVLYVGSK^{43}	3473.8980
4.26E-13	2.34	34	T^{33}KEGVLYVGSK^{43}	23	T^{22}KQGVAEAAGK^{32}	2376.2899
1.14E-12	0.67	12	A^{11}KEGVVAAAEK^{21}	97	K^{97}DQLGK^{102}	1897.0519
2.32E-12	1.24	45	T^{44}KEGVVHGVATVAEK^{58}	23	T^{22}KQGVAEAAGK^{32}	2720.4707
5.94E-12	1.20	96	T^{81}VEGAGSIAAATGFVKK^{97}	12	A^{11}KEGVVAAAEK^{21}	2815.5330
1.07E-10	0.52	43	T^{33}KEGVLYVGSKTK^{45}	12	A^{11}KEGVVAAAEK^{21}	2618.4529
5.04E-09	0.91	21	A^{11}KEGVVAAAEKTK^{23}	97	K^{97}DQLGK^{102}	2126.1946
2.29E-08	1.40	96	T^{91}VEGAGSIAAATGFVKK^{97}	23	T^{22}KQGVAEAAGK^{32}	2802.5126
1.52E-07	1.15	43	T^{33}KEGVLYVGSKTK^{45}	10	G^{7}LSKAK^{10}	2149.2357
2.52E-07	-3.62	34	T^{33}KEGVLYVGSK^{43}	12	A^{11}KEGVVAAAEK^{21}	2389.3103
5.32E-07	-2.34	60	T^{59}KEQVTNVGGAVVTGVTAVAQK^{80}	21	E^{13}GVVAAAEKTK^{23}	3395.8510
7.21E-07	0.58	12	A^{11}KEGVVAAAEK^{21}	12	A^{11}KEGVVAAAEK^{21}	2281.2528
7.33E-07	2.58	32	T^{22}KQGVAEAAGKTK^{34}	23	T^{22}KQGVAEAAGK^{32}	2484.3546
8.89E-07	0.99	21	E^{13}GVVAAAEKTK^{23}	10	G^{7}LSKAK^{10}	1842.0461

Δ: mass error for crosslink assignments; Res: crosslinked residue (sequence number) within corresponding proteins; Pr: protein; Underlined K in sequence is the cross-linked lysine.

*The crosslinked pair of residues were from two individual Ac-α-syn: ^{14}N-Ac-α-syn and ^{14}N-Ac-α-syn, ^{14}N-Ac-α-syn and ^{15}N-Ac-α-syn, ^{15}N-Ac-α-syn and ^{14}N-Ac-α-syn, or ^{15}N-Ac-α-syn and ^{15}N-Ac-α-syn. Detailed data processing procedure could be referred to Methods session.

Table S4 Identified cross-linked peptides of sample 1 between ^{15}N-Ac-α-syn and ^{14}N-Ac-α-syn in DOPS (protein: lipid= 1: 50, mol: mol).

**Score**	**Δ (ppm)**	**Res of Pr 1***	**Sequence of Pr 1***	**Res of Pr2***	**Sequence of Pr 2***	**Mass (Da)**
2.08E-16	0.45	32	Q^{24}GVAEAAGKTK^{34}	10	G^{7}LSKAK^{10}	1799.0151
2.67E-14	2.65	45	T^{44}KEGVVHGVATVAEK^{58}	23	T^{22}KQGVAEAAGK^{32}	2720.4707
4.22E-14	-2.67	45	T^{44}KEGVVHGVATVAEK^{58}	12	A^{11}KEGVVAAAEK^{21}	2733.4911
1.76E-12	-0.66	12	A^{11}KEGVVAAAEK^{21}	23	T^{22}KQGVAEAAGK^{32}	2268.2324
1.92E-09	-0.87	21	E^{13}GVVAAAEKTK^{23}	23	T^{22}KQGVAEAAGK^{32}	2298.2430
8.17E-09	1.29	21	A^{11}KEGVVAAAEKTK^{23}	10	G^{7}LSKAK^{10}	2041.1782

Δ: mass error for crosslink assignments; Res: crosslinked residue (sequence number) within corresponding proteins; Pr: protein; Underlined K in sequence is the cross-linked lysine.

*The crosslinked pair of residues were from two individual Ac-α-syn: ^{14}N-Ac-α-syn and ^{14}N-Ac-α-syn, ^{14}N-Ac-α-syn and ^{15}N-Ac-α-syn, ^{15}N-Ac-α-syn and ^{14}N-Ac-α-syn, or ^{15}N-Ac-α-syn and ^{15}N-Ac-α-syn. Detailed data processing procedure could be referred to Methods session.

Table S5 Identified cross-linked peptides of sample 2 between ^{15}N-Ac-α-syn and ^{14}N-Ac-α-syn in DOPS (protein: lipid= 1: 50, mol: mol).

Score	Δ (ppm)	Res of Pr 1*	Sequence of Pr 1*	Res of Pr2*	Sequence of Pr 2*	Mass (Da)
3.01E-15	-0.73	43	T^{33}KEGVLYVGSKTK^{45}	43	E^{35}GVLYVGSKTK^{45}	2726.5105
1.71E-11	2.06	96	T^{81}VEGAGSIAAATGFVKK^{97}	12	A^{11}KEGVVAAAEK^{21}	2815.5330
1.22E-10	-2.90	32	Q^{24}GVAEAAGKTK^{34}	97	K^{97}DQLGK^{102}	1884.0315
3.80E-08	-0.35	96	T^{81}VEGAGSIAAATGFVKK^{97}	10	G^{7}LSKAK^{10}	2346.3157
4.64E-07	0.38	23	T^{22}KQGVAEAAGK^{32}	23	T^{22}KQGVAEAAGK^{32}	2255.2120

Δ: mass error for crosslink assignments; Res: crosslinked residue (sequence number) within corresponding proteins; Pr: protein; Underlined K in sequence is the cross-linked lysine.

*The crosslinked pair of residues were from two individual Ac-α-syn: ^{14}N-Ac-α-syn and ^{14}N-Ac-α-syn, ^{14}N-Ac-α-syn and ^{15}N-Ac-α-syn, ^{15}N-Ac-α-syn and ^{14}N-Ac-α-syn, or ^{15}N-Ac-α-syn and ^{15}N-Ac-α-syn. Detailed data processing procedure could be referred to Methods session.

Table S6 Identified cross-linked peptides of sample 3 between ^{15}N-Ac-α-syn and ^{14}N-Ac-α-syn in DOPS (protein: lipid= 1: 50, mol: mol).

Score	Δ (ppm)	Res of Pr 1*	Sequence of Pr 1*	Res of Pr2*	Sequence of Pr 2*	Mass (Da)
4.55E-14	0.36	32	Q^{24}GVAEAAG$\underline{K}$TKEGVLYVGSK^{43}	21	A^{11}KEGVVAAAE$\underline{K}$TK^{23}	3429.8717
9.60E-14	0.25	32	Q^{24}GVAEAAG$\underline{K}$TK^{34}	10	G^{7}LS$\underline{K}$AK^{10}	1799.0151
1.05E-07	-1.97	45	T$^{44}\underline{K}$EGVVHGVATVAEK^{58}	43	E^{35}GVLYVGS$\underline{K}$TK^{45}	2841.5486
1.08E-07	1.16	23	T$^{22}\underline{K}$QGVAEAAGK^{32}	32	Q^{24}GVAEAAG$\underline{K}$TK^{34}	2255.2120
2.30E-07	-1.49	96	T^{81}VEGAGSIAAATGFV$\underline{K}$K^{97}	12	A$^{11}\underline{K}$EGVVAAAEK^{21}	2815.5330
5.89E-07	3.15	6	M^{1}DVFM$\underline{K}$GLSK^{10}	32	Q^{24}GVAEAAG$\underline{K}$TK^{34}	2351.2228

Δ: mass error for crosslink assignments; Res: crosslinked residue (sequence number) within corresponding proteins; Pr: protein; Underlined K in sequence is the cross-linked lysine.

*The crosslinked pair of residues were from two individual Ac-α-syn: ^{14}N-Ac-α-syn and ^{14}N-Ac-α-syn, ^{14}N-Ac-α-syn and ^{15}N-Ac-α-syn, ^{15}N-Ac-α-syn and ^{14}N-Ac-α-syn, or ^{15}N-Ac-α-syn and ^{15}N-Ac-α-syn. Detailed data processing procedure could be referred to Methods session.
